# Editorial: COVID-19: Challenges, opportunities and lessons for occupational health

**DOI:** 10.3389/fpubh.2022.1123920

**Published:** 2023-01-06

**Authors:** Min Zhang, Rokho Kim, Yonah Amster, Jorma Rantanen, Thomas H. Gassert

**Affiliations:** ^1^School of Population Medicine and Public Health, Chinese Academy of Medical Sciences/Peking Union Medical College, Beijing, China; ^2^Science Division, Department of Quality Assurance, Norms and Standards, World Health Organization, Geneva, Switzerland; ^3^School of Public Health, University of Haifa, Haifa, Israel; ^4^University of Helsinki, Helsinki, Finland; ^5^T.H. Chan School of Public Health, Harvard University, Boston, MA, United States

**Keywords:** COVID-19, health workers, occupational health, mental health, risk factors

## Introduction

We offer a collection of viewpoints from international submissions addressing the impact of the current pandemic on workers and their health.

As of end November 2022, the likely underestimated confirmed global COVID-19 death count was 6.7 million, and total confirmed cases were 650 million, among a global 8 billion population. It has exacerbated poverty and economic, social and political inequalities. The COVID-19 pandemic has incurred a devastating impact on workers globally, and has adversely affected worker rights, including those of the vast informal, migrant, temporary and unemployed workforce (1, 2).

In October 2021, *Frontiers* petitioned globally for studies of the impact of COVID-19 on occupational health to come up with lessons to prepare for future pandemics. Some key topics posted by the editorial panel for research included occupational health equity, international cooperation, medico-legal aspects, vulnerable workers, and a call for strategy and policy insights.

Since then, a total of 19 papers from 11 nations were published. Eleven papers focused on mental, physical and performance impacts of health workers, while three reported on non-healthcare front-facing services, education (teachers and students), and other sectors, respectively. One was a study of exposure risk indicators proposing a possible new job risk measure of Hospital Daily Admissions where other standard epidemiological indices might be sparse, as in low-resource settings.

Health workers were undoubtedly among the highest risk groups for adverse impacts. However, the risk of severe COVID-19 infection among health workers can be remarkably reduced by strict adherence to public health measures at the workplace as was reported by a recent study (3). This suggests that there is still much to be investigated and learned to inform preventive and control guidance for future pandemics, not only in health work but in all work settings.

A summary of the 19 articles follows. Most were cross-sectional descriptive studies based on questionnaire surveys or records review, though qualitative reports and opinions were included.

## Health workers

### Mental health

A survey from Iran by Karimi et al., of 170 hospital nursing staff caring for COVID-19 patients, using the Maslach Burnout Inventory, Turnover Intention and Michigan Organizational Assessment for Intent to Leave questionnaires, suggested that reduced personal accomplishment was a strong predictor of intent to leave. They recommended coping strategy counseling.

A report by Wang H. et al., sought to clarify stressful factors affecting health workers in temporary alternative care facilities in northeast China that handled hospital overflow during the COVID-19 pandemic. They identified five major factors with passive factors related to facilities design and active factors related to personal protective equipment (PPE) and counseling.

A systematic review of 12 studies of adverse physical and psychological impacts and adaptations of 121 COVID-19 ICU nurses (China 6, Turkey 2, Iran 2, USA 1, Spain 1) by Han et al., found that managers should support nurses with strategies integrating all aspects of the work and social environment to maintain workforce coping and satisfaction.

A survey from China of comparing a total of 1,000+ nurses and COVID-19 patients by Zhao et al., using the wellknown PHQ-9 (depression) and GAD-7 (anxiety) inventories found that nurses exhibited significantly more depression than patients, while patients were more anxious than nurses. Over 95% of these frontline nurses and COVID-19 patients reported having not received pre-pandemic counseling, and such counseling was recommended.

Daryanto et al. surveyed 1,077 health workers in an urban area of Java Indonesia and found that one-fifth suffered burnout, most strongly associated with young age and long work hours.

An online descriptive psychological survey from Japan by Sawamura et al., of 4,418 occupational therapists (OT) in two work domains—physical and mental health services—assessed the prevalence of anxiety, depression, insomnia and loneliness and found decreases in OT care quality with the main factor being depression in the physical health OT service sector, and insomnia in the mental health OT sector.

### Risk factors

From northern Pakistan, Manzoor and Alomari utilized a Capability Opportunity Motivation-Behavioral model (COM-B) to investigate factors among 9,000 dentists that determine degree of adherence to COVID-19 Standard Operating Procedures (SOPs) for dental surgical procedures. They suggested the importance of providing increased holistic support through infrastructure, facilities, financing, training and PPE to increase adoption of COVID-19 SOPs.

A qualitative review of COVID-19 hospital ward nurses in Iran by Mokhtari et al. identified four risk categories: sudden unknown threat exposure; exaggerated stress; feeling of being in an unequal war; and need to increase efforts to confine the threat to maintain good ethical and clinical decision-making. Their concerns were thought to be rooted in organizational and governmental issues.

A Patient Safety Culture Survey using a six-dimension safety attitudes questionnaire of 706 COVID-19 health workers in Taiwan by Wang S. J. et al., intended to address improvement in patient outcomes and health worker risks, revealed that key risk factors affecting patient safety were health worker emotional exhaustion (EE) and work-life balance (WLB) disruption. Government interventions that decreased workload to reasonable levels and that enhanced communication, improved health worker attitudes from negative to positive on safety climate, job satisfaction and perception of management. EE and WLB also improved.

Over 4 weeks at a hospital outpatient clinic in China, Zhang et al. surveyed body temperature and symptoms by questionnaire of all, >60,000, patients. They recommended increasing strategies for patient screening to improve prevention of health worker COVID-19 infection risk.

Compliance with IPC best practices by 600 health workers in Malaysia was assessed by Mohamad et al., using the WHO Interim Guidance questionnaire on exposure risk assessment and management for health workers. They reported a 63.7% compliance rate (all responses “always”), leaving a significant >36% of health workers not compliant. The authors recommended intervention and monitoring programs for IPC and OH programs such as an OH committee.

A creative study from France, Valter et al., proposed a possible new standard for COVID-19 exposure risk for communities and work places (JEM, job exposure matrices) that we already know locally to include these four: ICU % occupancy; reproductive number (R_0_), COVID-19 test positivity rate; and number of positive cases per population reference. These epidemiological risk estimates are often difficult to truly compare. The authors proposed a fifth JEM called Daily Hospital Admissions (DHA) on a population level that can be applied to specific local job titles. They suggested DHA might be particularly useful in low-resource settings where data are lacking for other JEMs.

### Hand dermatitis and workplace violence

Clinical and hierarchy of control interventions were the focus of presumptive irritant contact hand dermatitis among a cohort of 21 health workers in a Singapore hospital related to ABHR (alcohol-based hand rub) product use averaging 50 times daily, and possibly glove use including latex, reported by Loi et al. Clinical outcomes were followed by the hospital occupational health service doctors over several weeks. While some health workers were variably relegated to topical treatment and to temporary work restriction, or to modified duties to reduce exposures, ~80% reported improved symptoms, some with full resolution. Authors recommended milder ABHRs and if needed temporary job modification, with consideration of elimination of latex gloves and further evaluation.

Patient and visitor violence (PVV), a kind of Work Place Violence (WPV), toward health workers is common and during COVID-19 was studied in a survey of 754 health workers in China by Guo et al., who reported doctors were at 5.3 times higher risk of physical PVV compared with nursing staff. The authors identified that security measures are very important to protect health workers from PVV, and recommended comprehensive IPC and WPV programs.

## Workers of other sectors

A qualitative interview by Wei H. et al., of 11 frontline workers in 6 companies in the “logistics” sector in the UK (takeaway and food delivery, goods delivery, home appliance installation, and tech services) identified drivers of and obstacles to rapid implementation of Public Health Non-Pharmaceutical Interventions and Occupational Hygiene Hierarchy of Controls including COVID-19 testing. They recommended a “rapid response model” to address IPC and RMM (risk mitigation measures).

An online survey of 27,036 workers in Japan (50% desk work, 25% laborers, and 25% customer communicators) by Tesen et al., suggested that loneliness should be considered a risk for sleep problems and that family and friend support may have a modifying effect on sleep disturbance.

Canadian teachers were surveyed cross-sectionally using the WHO Disability Assessment Schedule-2.0 by Serrano et al., on their perception of COVID-19 impact on work function. Six functional domains (cognition, mobility, self-care, getting along, life activities, participation) were assessed as either unchanged, worse, or better. Risk factors included pre-existing inequality and mental health challenges as predictors. Educators reported worsening of work function from the start of the COVID-19 pandemic. Mental health challenges and pre-existing inequality were considered predictors of pandemic-related performance difficulties. Recommendations included worker telehealth counseling services and policies for overall self-health promotion.

A descriptive study by Wei C.-F. et al., of 780 health workers and customer facing workers at a community COVID-19 testing center in the USA reported a four-fold risk of COVID-19 infection in health workers and a two-fold risk of COVID-19 infection among customer facing workers. This was compared with non-customer facing workers.

## Opportunities

Sara from the US outlined three opportunities for lasting public health change and future pandemics crises: tele-healthcare, remote work and remote education, and vaccinations.

## Lessons and recommendations^1^

Nineteen studies were published in 2022 in the Research Topic co-edited by us. Most were cross-sectional surveys, a majority on health workers, and there were a few other work group studies with recommendations for future prevention.

Contributions were very enlightening. We find however that there is still a paucity of studies to help explain the hideous ways of pandemics among working populations. As such, there is a need to continue pandemic research globally with regard to workers in all sectors. It clearly has a place among health workers but also among so many other vulnerable worker sectors who lack adequate individual means of infection prevention and control.

Studies published in this Research Topic indicate that pro-active workplace implementation of evidence-based public health and occupational health measures regarding principles of IPC and of occupational hygiene hierarchy of controls, including vaccinations and honest media communication, is the key to workplace pandemic preparedness and trust. We wish to add that the implementation of preventive basic occupational health services especially for vulnerable workplaces and communities in low- and middle-income countries, with proper foresight, planning and finance, will contribute to mitigating future pandemics.

For the world to be better prepared for future pandemics, the followings could be emphasized: (1) A global need for better mechanisms for prediction, risk assessment, and preparedness for pandemics, paying attention to the workers, working environment and occupational hygiene of the most vulnerable sectors; (2) Strategies, policies, and programs for earliest possible warnings and actions for eliminating the sources of local epidemics and preventing them from growing to a pandemic, taking into account of the workplace, which is often on the frontline of epidemic risks and may also be a distributor of risks to the rest of society (health sector, food industries, service sectors, schools, etc.); and (3) Sufficient and well-maintained resources and reserves at the workplace, local community, national and global levels for effective prevention and management of epidemic risks, including juridical, organizational, material, information, and human resources.

In the global context of international cooperation, the most equitable approach to mitigating the future pandemics is the universal provision of preventive occupational health services for all workers, especially for the vulnerable, within the framework of the UN Sustainable Development Goals (SDGs) on Universal Health Coverage (SDG-3) and Decent Work (SDG-8).

## Author contributions

MZ drafted the first manuscript and other co-editors provided comments. TG, JR, and YA contributed to formulating key lessons. TG and RK prepared the final version which was approved by all co-authors. All authors contributed to the article and approved the submitted version.

## Footnotes

^1^Recommendations in this editorial are of the opinion of co-editors, not of their employers by any means.
